# A Biosafety Agenda to Spur Biotechnology Development and Prevent Accidents

**DOI:** 10.1089/hs.2016.0095

**Published:** 2017-02-01

**Authors:** Gigi Kwik Gronvall

Biosafety—keeping laboratory workers, the community, and the environment safe—is a crosscutting need for all research activities, as well as an important research and policy area with medical, political, and security consequences. As such, it requires a dedicated plan for the US government. One reason biosafety is attracting more attention now is because it may be a limiting factor in the development of advanced biotechnologies. Many potential new biotechnology applications with biomedical and economic implications are intended to be used outside the laboratory and released into the environment, so their safety cannot rely on traditional laboratory containment. They are meant to be “outside.” Applications such as mosquito control, agriculture, pollution remediation, mining, biofuels, medications that use synthetic organisms to treat gastrointestinal diseases, or even the re-creation of extinct animals require synthetic organisms to be in the environment, where they can interact with other living things. These applications may be tremendously beneficial and may spur economic development, but if biosafety risks are not addressed and carefully thought through, they could yield unintended and accidental consequences.

Biosafety has become more politically important in recent times as well, particularly after multiple high-profile safety failures at federal and military laboratories involving smallpox, anthrax, and flu. In 2014, decades-old glass vials containing live variola (smallpox) virus were discovered at the US Food and Drug Administration (FDA). Smallpox was declared eradicated in 1980 and is supposed to be held only under tightly controlled conditions at the Centers for Disease Control and Prevention (CDC) and a designated Russian laboratory. Given that decades have passed and the samples were not either transferred or disposed of, this incident was indicative of poor inventory management. In 2015, it was discovered that the US Army Dugway Proving Ground had shipped samples containing live anthrax to centers not registered to work with it. These samples were incompletely irradiated, and some vials still had live anthrax spores. Additional potential exposures of anthrax and noncirculating influenza at the CDC occurred as well.

A series of comprehensive reforms affecting biosafety have already begun under the Obama Administration. Reform was motivated by laboratory incidents, but also by mounting concerns about the increased power and democratization of biotechnology: Consequential bio-errors may occur more frequently if more people are able to work on infectious diseases. If a laboratory accident occurs with a transmissible pathogen, the consequences could spread well beyond the laboratory—and beyond national borders. Many of these much-needed government reforms to biosafety are still in the process of being implemented. Yet, there are additional, new opportunities that will be important to pursue to increase safety in the coming years, including dedicating research funds to biosafety; expanding biosafety training into new environments, to reach the growing populations of people who are performing biological work; and leading the world to develop international biosafety norms.

## Recommendations

➢ **Biosafety information is needed in order to advance biotechnology as well as to prevent laboratory failures, but little is currently being funded or performed. For safety, economic, and political reasons, the US government needs to fund biosafety research.**

Advanced biotechnology products will not be of any medical, manufacturing, or economic benefit if they can't be proven to be safe. In order to realize the benefits of biotechnology, there is a stark need for specific safety studies for advanced biotechnology products, including synthetic organisms used for environmental remediation or medicines that could theoretically remain in the environment after they are no longer needed, so that they can be safely used outside traditional laboratory settings.

One approach to containment outside the laboratory is called “intrinsic” biosafety—that is, the biosafety is built into the organism, so that the synthetic organisms can't escape boundaries that are set for them, even if they are outside of the laboratory. Some forms of intrinsic biosafety involve engineering organisms so that they are not able to survive without specific human intervention, such as by supplying a nutrient that is essential for life. If the nutrient is not supplied, the synthetic organism will die, and it will stay “contained” in the area where that essential nutrient is supplied. Early attempts to develop intrinsic biosafety were often unsuccessful and had high “escape” rates, because they relied on a single point of failure. Newer intrinsic biosafety approaches are more subtle and rely on combinatorial complexity, which drastically reduces the rates of failure and escape—that is, many safety systems would have to fail before the organism could escape. Some intrinsic biosafety work has been initiated by DARPA, but much more is needed if promising biotechnology advances are going to be translated into real products.

There is also a great need for data to reduce and prevent accidents in the laboratory. The need for data that describe laboratory incident and accident rates is well understood—How can problems be addressed if the incidence and causes for safety failures are not known?—and efforts are under way in the US government to set up a no-fault reporting system modeled on one used in the aviation industry to reduce accidents.

However, more information related to biosafety is needed to set policy in this area, which requires research: There is a need for *procedural studies* (such as the proper protocols to inactivate anthrax spores, which equipment works best for a given protocol, which personal protective equipment [PPE] works best to protect the laboratory worker); *behavioral studies* (how best to instill a safety culture in the laboratory; what is the best training material for each skill level of scientist; how can laboratories be inspected in such a way as to be supportive and improve safety over time; how to promote safe practices in routinized biological laboratory environments); and *comparative studies* (to determine the safety level for different laboratory practices, engineering, laboratory set-ups, and equipment).

➢ **Biological research has become democratized, but biosafety training has not. The US government needs to expand biosafety training and access to biosafety advice to the people who require it.**

Typically, laboratory skills including biosafety are developed over years of training and mentorship in biological research laboratories. Yet, with the advent of new biotechnologies that are relatively inexpensive and able to be pursued outside of a traditional laboratory, more people have begun to do biology research, including amateurs: There is an active, international DIY Bio movement. There have been some outreach programs set up to bring biosafety training to these new practitioners. Community laboratories, a source for education programs, have safety requirements, and there is an “ask a biosafety expert” program freely available on the DIY Bio website. But much more is needed, and as the technologies get more powerful, this problem is likely only to expand.

CRISPR, the revolutionary new gene editing tool, is available to amateurs all over the world in a kit that costs less than $150. There is a pressing need to adapt biosafety training so that the people who need safety advice have an opportunity to receive it. There are also security benefits for reaching out into the communities where this work is performed; the FBI has taken this approach in the WMD Directorate to institute a “see something, say something” campaign for biologists.

➢ **Laboratory accidents with highly transmissible agents anywhere in the world have the potential to become a public health emergency of international concern. The United States should work toward international norms for biosafety so that all nations appropriately oversee, fund, and train to prevent accidents where possible and mitigate when necessary.**

Most accidents in biocontainment laboratories are limited to the researchers involved and possibly their close contacts. While these accidents are unfortunate events that may have severe consequences for those who are affected, they would not typically become matters of national or international concern. However, laboratory-acquired infections with particularly transmissible pathogens could have consequences that go well beyond the laboratory, beyond borders, and would constitute a threat to national and global security. Consequential laboratory accidents have happened before, but thankfully with limited impacts on international human health: For example, in 2003-04, there were multiple laboratory-acquired infections with the SARS virus, but a quick response halted transmission before the virus could spread widely.

There is a great deal of technical guidance for researchers and institutions to achieve high levels of safety, to train workers, and to foster a laboratory environment that holds safety as a priority. However, national-level biosafety norms could provide reassurance to other nations that research with highly transmissible pathogens is performed with appropriate and sufficient safety systems. It is almost certain that such norms would develop in the aftermath of a laboratory accident, so these commonsense measures should be put in place now. For example, it would be helpful to know that all such research is taking place in countries where there are national standards for the work, including for equipment maintenance, worker safety training, health monitoring, surveillance, and other myriad activities to help keep the researchers and the larger public safe, and that the nation has an adequate surveillance system in place to identify and limit potential outbreaks that could result from such accidents.

Without national-level standards and expectations for biosafety, there will remain insufficient incentives to commit the resources required to achieve high levels of biosafety in individual laboratories and institutions. Absent biosafety norms, the United States will not have confidence that all necessary steps are being taken in other nations to prevent a high-consequence laboratory accident from occurring, or to limit its consequences.

